# A close look at the biology of SARS-CoV-2, and the potential influence of weather conditions and seasons on COVID-19 case spread

**DOI:** 10.1186/s40249-020-00688-1

**Published:** 2020-06-26

**Authors:** Kamoru A. Adedokun, Ayodeji O. Olarinmoye, Jelili O. Mustapha, Ramat T. Kamorudeen

**Affiliations:** 1 Department of Oral Pathology, King Saud University Medical City, Riyadh, Saudi Arabia; 2 Centre for Control and Prevention of Zoonoses, University of Ibadan, Ibadan, Nigeria; 3 Engineer Abdullah Bugshan Research Chair for Dental and Oral Rehabilitation (DOR), King Saud University, Riyadh, Saudi Arabia; 4 Biological Science Department, University of Alberta, Edmonton, Alberta T6G 2E9 Canada; 5 Public Health Department, University of South Wales, Pontypridd, UK

**Keywords:** Case spread, COVID-19, Season, Emergency preparedness, Human immunity, Infection control, SARS-CoV-2

## Abstract

**Background:**

There is sufficient epidemiological and biological evidence of increased human susceptibility to viral pathogens such as Middle East respiratory syndrome coronavirus, respiratory syncytial virus, human metapneumovirus and influenza virus, in cold weather. The pattern of outbreak of the coronavirus disease 2019 (COVID-19) in China during the flu season is further proof that meteorological conditions may potentially influence the susceptibility of human populations to coronaviruses, a situation that may become increasingly evident as the current global pandemic of COVID-19 unfolds.

**Main body:**

A very rapid spread and high mortality rates have characterized the COVID-19 pandemic in countries north of the equator where air temperatures have been seasonally low. It is unclear if the currently high rates of COVID-19 infections in countries of the northern hemisphere will wane during the summer months, or if fewer people overall will become infected with COVID-19 in countries south of the equator where warmer weather conditions prevail through most of the year. However, apart from the influence of seasons, evidence based on the structural biology and biochemical properties of many enveloped viruses similar to the novel severe acute respiratory syndrome coronavirus 2 or SARS-CoV-2 (aetiology of COVID-19), support the higher likelihood of the latter of the two outcomes. Other factors that may potentially impact the rate of virus spread include the effectiveness of infection control practices, individual and herd immunity, and emergency preparedness levels of countries.

**Conclusion:**

This report highlights the potential influence of weather conditions, seasons and non-climatological factors on the geographical spread of cases of COVID-19 across the globe.

## Background

The coronavirus disease 2019 (COVID-19) has become a global pandemic and a threat to the public health systems across the world. Much about the dynamics of the transmission of the causative agent of COVID-19, the novel severe acute respiratory syndrome coronavirus 2 (SARS-CoV-2), is still unknown. However, it has been established that SARS-CoV-2 is an enveloped positive-sense single stranded RNA virus (+ssRNA) [1]. The lipid bilayer envelope, membrane proteins and nucleocapsid of enveloped viruses are elements of the structural biology of these pathogens that confer protection to them outside their host cells [2]. However, the lipid bilayer within the cell membrane of enveloped viruses consists of cholesterols and phospholipids, and will only allow the virus to survive for a limited time outside the host cell environment. Hence, to survive, enveloped viruses need to be transferred directly from one host to another, as quickly as possible. A critical look at the epidemiology of some enveloped viruses will reveal the influence of seasons on their activities. The incidence of infections caused by enveloped viruses such as respiratory syncytial virus (RSV), influenza virus and human metapneumovirus (hMPV) is higher when environmental temperatures are lower but RSV and influenza virus cases are limited to the cold winter months while hMPV cases are seen throughout the year and peak in later winter and spring [3]. Based on the structural, biological and biochemical similarities of these ‘cold-season’ viruses and SARS-CoV-2, it could perhaps be the situation that the current rampant spread of COVID-19 cases will wane during summer months in the northern hemisphere when the weather becomes warmer. This report takes a critical look at the potential influence of weather conditions on the geographical distribution and transmission of COVID-19, amidst the consideration of other non-climatological factors.

### Epidemiology and transmission of COVID-19

COVID-19 is a pandemic and global health emergency caused by SARS-CoV-2, previously called 2019 novel coronavirus (2019-nCoV) [3]. SARS-CoV-2 belongs to the virus order Nidovirales, family Coronaviridae, genus *Betacoronavirus* (enveloped, positive-sense, single-stranded RNA viruses that are zoonotic) [3] and sub-genus *Sarbecovirus,* same as SARS-COV the aetiologic agent of the 2003 SARS outbreak with likely origin in bats, and likely involvement of another (intermediate) host animal such as the pangolin [3, 4].

Human-to-human transmission of SARS-CoV-2 is primarily via respiratory droplets and by direct contact with infected people and indirect contact with contaminated surfaces and objects [3]. The mean incubation period of SARS-CoV-2 infection varies from 5 to 6 days and the range 1–14 days [3]. All age groups of humans are susceptible to SARS-CoV-2 infection but at particularly higher risk are the aged, immunocompromised individuals, and people with chronic underlying diseases. The disease manifests as a flu-like (respiratory) illness characterized by fever, chills, sore throat, dry cough, expectoration, dyspnoea, fatigue, headache, myalgia or arthralgia, and less commonly hemoptysis, conjunctival congestion and gastrointestinal tract involvement i.e. nausea or vomiting and diarrhoea [3]. The case fatality rate (CFR) of COVID-19 has been increasing and is currently higher than what was earlier reported (Table 1) [5].

**Table 1:** Epidemiological features of COVID-19 and other similar viral infections

Disease	COVID-19	Flu	RSV infection	SARS
Viral agent	SARS-CoV-2	Influenza virus	RSV	SARS-CoV
Transmission	Airborne droplet	Airborne droplet	Airborne droplet	Airborne droplet
Infection period	N/A	fall and winter	winter	Spring
Basic reproduction number (*R*_o_)	2–2.5	1.7	3.5	2–5
Case fatality rate (CFR)	~ 7.1%^a^	0.05–0.1%	4.6%	9.6–11%
Incubation time	2–14 days^b^	1–4 days	4–5 days	2–7 days
Hospitalization rate	20%	2%	2.1%	Most cases
Annual infected (Global)	N/A	~ 1 billion	64 million	8098 (2003)

*COVID-19* Coronavirus disease 2019

*SARS-CoV-2* Severe acute respiratory syndrome coronavirus 2

*RSV* Respiratory syncytial virus

^a^Data: Updated by European Centre for Disease Prevention and Control (ECDC) on May 122 020)

^b^Median time (x͂) = 5–6 days

Nevertheless, the majority (80%) of COVID-19 patients show a mild form of the disease with or without pneumonia, and the remainder (20%) that typically represent the more susceptible population segments such as infants, the elderly and people with pre-existing conditions involving diabetes, hypertension, coronary heart disease and chronic obstructive pulmonary disease are more likely to experience a more severe form of the disease that is characterized by respiratory failure, septic shock, and or multiple organ dysfunction or failure [3]. The global human death toll due to COVID-19 has continued to rise and has outstripped those due to the previous epidemics caused by severe acute respiratory syndrome (SARS) and the Middle East respiratory syndrome, combined [6].

### Structural biology of SARS-CoV-2 and other enveloped viruses

**Fig. 1:**
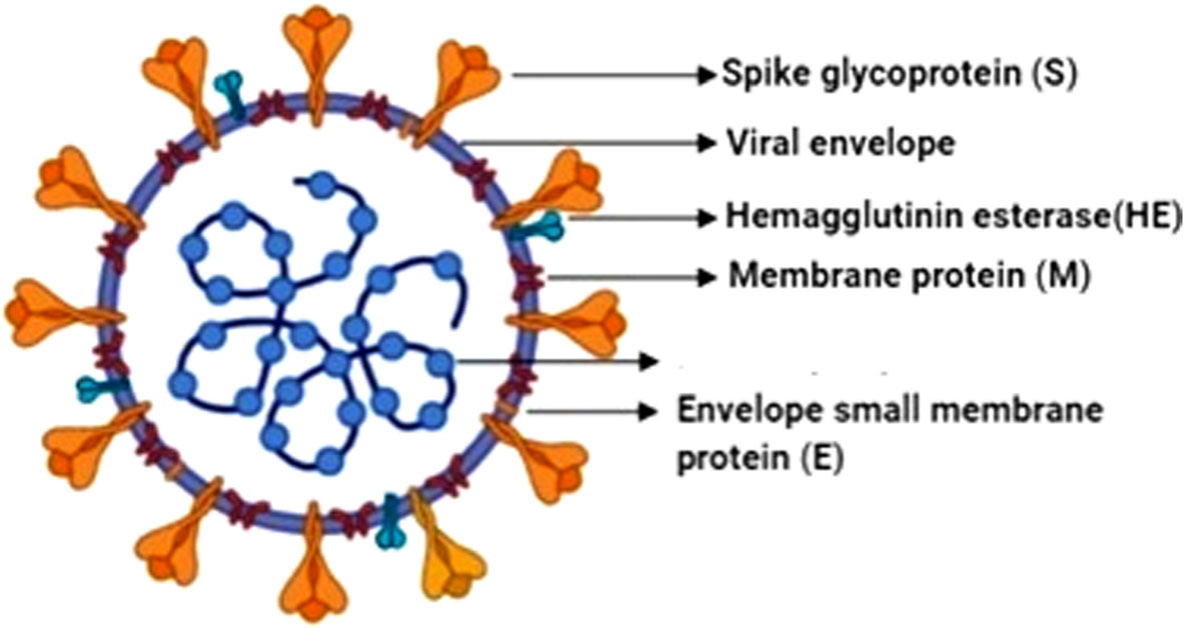
The structural features of coronavirus SARS-CoV-2 and its main structural proteins

The genome of SARS-CoV-2 is similar to that of other coronaviruses and has four genes that encode the following structural proteins – the S (spike), E (envelope), M (membrane), and N (nucleocapsid) proteins (Fig. 1). The N protein encapsulates the RNA genome (GenBank accession number: MN908947) that measures 29 903 nt in length while the other proteins (the S, E, and M) together comprise the viral envelope [1]. The presence of a lipid bilayer envelope makes a majority of enveloped viruses to be susceptible to destruction upon exposure to dry heat, detergents and organic solvents [2]. Thus, enveloped viruses such as SARS-CoV-2 may only survive outside host environments for a limited time and characteristically need to be transferred directly from one host to another to continue to survive. It is with the spike proteins that SARS-CoV-2 attaches and gains entry into human cells using specific receptors, especially the angiotensin converting enzyme 2 (ACE2) receptors on the membrane surface of human cells, to which the higher affinity of the spike protein has been demonstrated [7].

### Geographical spread and potential seasonality of human cases of COVID-19

COVID-19 cases have been reported in 208 countries/territories in six continents and caused over 285 760 deaths globally [5]. Cold temperatures favor the survival of cold viruses and reduce immunity in humans and animals by altering the cellular and molecular defenses against pathogens in the upper respiratory tract [8]. Some common colds experienced during winter and spring in countries of the northern hemisphere, are caused by viruses from the same family. Majority of these viruses are called cold-viruses and they have a cyclical pattern of occurrence in what is known as “flu season”. Flu viruses are widespread during the fall and winter months and peak between December and May [9]. Worldwide, cases of human morbidities and mortalities due to COVID-19 have continued to rise in the “flu season” but COVID-19 is not the flu. Data obtained from the National Meteorological Centre of China and the Hong Kong Observatory (http://gb.weather.gov.hk/), China, show that the outbreak of COVID-19 occurred during the cold season in the country, similar to the timing of the outbreak of the previous SARS epidemic. It was recently reported that 70.2% of people that were positive for COVID-19 and as much as 79.4% of COVID-19 deaths throughout Italy occurred in the northern provinces namely Lombardi, Emilia Romagna, Veneto and Piemonte, the same areas from were the first cases of the disease were recorded in the country [10]. However, according to the report, the skewed distribution of COVID-19 mortalities in Italy was not due to the colder weather of the north alone but likely, an interplay of factors including “demographics and health, societal customs and epidemic-specific attitudes, environmental factors and administrative issues” [10].

## Conclusions

**Fig. 2:**
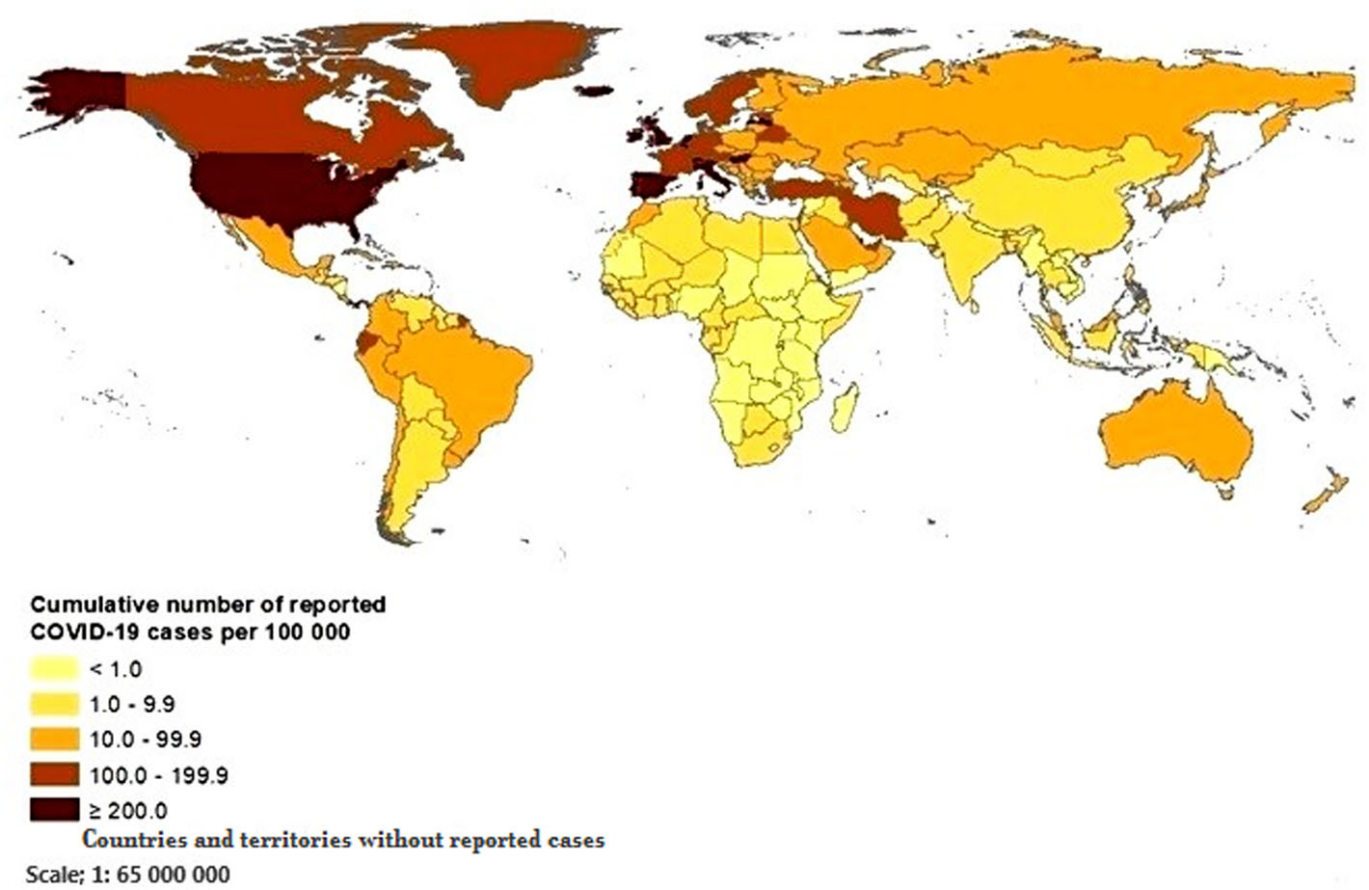
Global distribution of cumulative number of reported COVID-19 cases per 100 000 population (Source: European Centre for Disease Control and Prevention (ECDC); accessed 12 May 2020

Till date, the hotspots of COVID-19 have been more prevalent in the northern hemisphere with the worst affected countries being the United States (80 684 deaths), United Kingdom (32 141 deaths), Italy (30 739 deaths) and Spain (26 744 deaths) while in comparison, countries south of the equator have in general recorded fewer deaths (Fig. 2). It remains to be seen if COVID-19 will continue to spread unremittingly during summer in the northern hemisphere when environmental temperatures will start to rise, or whether the overall case numbers and mortalities in the warmer countries of the southern hemisphere will remain comparatively lower. The answers we seek may not be unconnected to the structural biology of SARS-CoV-2, especially as it relates to the transmission of this novel virus. More studies are required to identify and characterize the potential seasonality of COVID-19 cases as the current global pandemic continues to unfold.

## Data Availability

Not applicable.

## Abbreviations

+ssRNA: Positive-sense single stranded RNA virus
2019-nCoV: 2019 novel coronavirus
ACE2: Angiotensin converting enzyme 2
COVID-19: Coronavirus disease 2019
hMPV: Human metapneumovirus
RSV: Respiratory syncytial virus
SARS-CoV-2: Severe acute respiratory syndrome corona virus type 2
